# Comparing the Quality of Patient Recovery After Open Appendectomy Under General Versus Spinal Anesthesia: Prospective Cohort Study

**DOI:** 10.2196/86273

**Published:** 2026-06-25

**Authors:** Negesse Zurbachew Gobezie, Temesgen Birlie Asmare, Kumlachew Geta Belete, Gezahagn Demsu Gedefaw, Biruk Demissie, Abere Gebru Abuhay, Belayneh Jejaw Abate, Birhanu Mengist Munie, Wubet Tazeb Wondie, Habtie Bantider Wubet

**Affiliations:** 1 Department of Anesthesia, School of Medicine, College of Health Science, Debre Tabor University, P.O. Box 272, Debre Tabor, Ethiopia; 2 Department of Neonatal Health Nursing, School of Nursing, College of Medicine and Health Sciences, and Specialized Hospital, University of Gondar, Gondar, Ethiopia; 3 Department of Environmental Health, College of Health Science, Debre Tabor University, Debre Tabor, Ethiopia; 4 Department of Pediatrics and Neonatal Nursing, College of Health Sciences, Debre Tabor University, Debre Tabor, Ethiopia; 5 University of Gondar Comprehensive Specialized Hospital, Gondar, Ethiopia; 6 Department of Psychiatry, College of Health Science, Debre Tabor University, Debre Tabor, Ethiopia; 7 Department of Pediatrics and Child Health Nursing, School of Health Sciences, College of Medicine and Health Sciences, Bahir Dar University, Bahir Dar, Ethiopia

**Keywords:** appendectomy, anesthesia, surgery, outcome, recovery, postoperative

## Abstract

**Background:**

Open appendectomy is commonly performed under either general or spinal anesthesia. Postoperative quality of recovery, a multidimensional patient-centered measure of outcomes after surgery, is affected by the choice of anesthesia technique.

**Objective:**

The objective of our study was to compare the effects of general and spinal anesthesia on the immediate postoperative quality of recovery in adults undergoing open appendectomy.

**Methods:**

In this prospective cohort study, 74 patients were assigned to either the general (n=37) or the spinal anesthesia (n=37) groups. The primary outcome was the total Quality of Recovery-15 score measured 24 hours postoperatively. Secondary outcomes included postoperative pain scores, analgesic consumption, incidence of postoperative nausea and vomiting, time to first oral intake, time to first ambulation, and length of hospital stay. Intergroup comparisons were performed using the chi-square test, Fisher exact test, independent *t* test, or Mann-Whitney *U* test, as appropriate.

**Results:**

Patients in the spinal anesthesia group had significantly higher Quality of Recovery-15 scores than those in the general anesthesia group (*P=.*001). They also exhibited lower pain scores at 1, 2, 6, and 12 hours postoperatively (*P<.*001), reduced consumption of diclofenac and tramadol, and a decreased incidence of postoperative nausea and vomiting (*P<.*001). The time to first mobilization, time to first oral intake, and length of hospital stay were also significantly lower in the spinal anesthesia group (*P<.*001).

**Conclusions:**

Spinal anesthesia is the preferred anesthesia technique for patients undergoing open appendectomy. It may provide improved postoperative recovery, with lower pain scores and improved analgesia. It may also reduce the need for additional analgesics, decrease the incidence of nausea and vomiting, and improve intrahospital patient recovery trajectories.

## Introduction

Acute appendicitis, defined as inflammation of the vermiform appendix, remains one of the most common causes of acute abdomen and emergency abdominal surgery worldwide [1,2]. Current epidemiological data indicate an increasing incidence of this condition in both middle-income and high-income countries [3,4]. Although laparoscopic appendectomy is increasingly used in high-resource settings [5,6], open appendectomy remains the predominant approach in resource-limited settings and is performed under either general anesthesia (GA) with endotracheal intubation or spinal anesthesia (SA) [7-9].

GA is associated with several potential adverse effects, including airway complications during induction or extubation, hemodynamic instability, aspiration, and postoperative nausea and vomiting (PONV) [10-12]. SA has emerged as a viable alternative to GA, with increasing use in abdominal surgeries performed below the umbilicus, including open appendectomy [7,8,13,14]. The use of SA for abdominal surgeries is associated with superior postoperative pain control, fewer cardiorespiratory adverse events, and a lower incidence of PONV [15-17]. Nevertheless, these individual outcomes do not completely reflect the patient’s overall recovery from anesthesia and surgery [18]. Consequently, evaluating the quality of recovery (QoR) from the patient’s perspective has emerged as a critical metric for assessing the impact of anesthesia and surgery on overall patient recovery and well-being [19].

Postoperative QoR is a multidimensional outcome measure that encompasses physical comfort, emotional state, psychological support, physical independence, pain, and return to normal activities [20,21]. It has emerged as a holistic and patient-centered measure of surgical and anesthetic success compared to traditional, isolated metrics such as pain scores, duration of surgery, duration of anesthesia, length of hospital stay, and complication rates [22-24].

In addition to the surgical-related and patient factors such as age, comorbidities, nutritional status, and frailty status, the choice of anesthetic technique significantly influences postoperative outcomes and QoR [20]. Several studies have compared GA and SA across different surgical procedures and have frequently demonstrated the benefits of SA in terms of pain control, PONV, and length of hospital stay [25,26]. However, limited data exist regarding the impact of anesthetic techniques on postoperative QoR in patients undergoing open appendectomy.

Therefore, this study primarily aimed to compare the effects of GA and SA on immediate postoperative QoR in adult patients undergoing open appendectomy. The secondary objective of this study was to compare postoperative pain scores, analgesia consumption, incidence of PONV, and immediate intrahospital recovery trajectories such as time to first oral intake, first ambulation, and length of hospital stay. The findings of this study may provide evidence to guide anesthesia decision-making and optimize patient-centered outcomes in the surgical care for patients undergoing open appendectomy.

## Methods

### Overview

An institution-based prospective cohort study was conducted at Debre Tabor Comprehensive Specialized Hospital from December 1, 2024, to June 6, 2025. The hospital is located in Debre Tabor town, South Gondar Zone, Amhara Region, Northwest Ethiopia. It is a governmental hospital in Debre Tabor town that serves a population of approximately 3 million people. The hospital provides gynecologic, obstetric, orthopedic, and general abdominal and urologic surgical procedures on an elective and emergency basis. It has 2 operating rooms and 1 dedicated surgical ward with 32 beds, specifically allocated to patients undergoing general surgery.

### Study Population

Adult surgical patients aged ≥18 years with American Society of Anesthesiologists physical status classification I or II, diagnosed with acute appendicitis, and who underwent open appendectomy were included in our study. Patients were excluded if they met any of the following criteria: American Society of Anesthesiologists physical status ≥III, pre-existing neuropsychiatric disorders impairing effective communication, periappendiceal abscess or generalized peritonitis requiring midline laparotomy, intraoperative discovery of additional pathologies necessitating concurrent surgical procedures, inadequate spinal block necessitating conversion to GA during the operation, or occurrence of unexpected intraoperative catastrophic events requiring postoperative admission to the intensive care unit.

### Sample Size Determination and Sampling Procedure

The sample size for this study was calculated using a standard formula to compare means between 2 independent groups. The primary outcome of our study was the mean difference in the Quality of Recovery-15 (QoR-15) score at 24 hours postoperatively: n=(σ_1_^2^+σ_2_^2^)(Z_β_+Z_α/2_)^2^/(µ_1_–µ_2_)^2^, where n is the required sample size for each group; σ_1_ and µ_1_ are the SD and mean of the first group (SA), respectively; σ_2_ and µ_2_ are the SD and mean of the second group (GA), respectively; *Z_α_*_/2_ is the critical value for the desired confidence level; and Z_β_ is the critical value for the desired statistical power.

As no similar study had been conducted in the study setting, parameter estimates for sample size calculation were obtained from a pilot study (n=20) conducted 1 month before the actual data collection period. In the pilot study, the mean QoR-15 scores for the SA and GA groups were 123.9 (SD 22.4) and 111.2 (SD 14.6), respectively. Using a 95% significance level (Z_α/2_=1.96) and 80% power (Z_β_=0.84): n=[{(22.4)^2^+(14.6)^2^}(1.96+0.84)^2^]/(123.9–111.2)^2^=34.7≈35. After adding a 5% nonresponse rate, the final sample size was 37 participants per group, resulting in a total of 74 participants included in the study. Subsequently, all consecutive patients who met the inclusion criteria were included in the study until the required sample size was achieved.

### Data Collection Procedure, Data Collection Tools, and Measurements

A structured questionnaire was prepared to collect information on sociodemographic characteristics, comorbidities, medication use, and anesthesia- and surgery-related variables during the preoperative, intraoperative, and postoperative periods. The original questionnaire was developed in English and was subsequently translated into Amharic. To ensure linguistic accuracy and maintain the original content, the questionnaire was back-translated into English by 2 bilingual language experts fluent in both English and Amharic. Data were collected through chart reviews and direct interviews with participants conducted by anesthetists and nurses who were not directly involved in patient management.

In the study setting, anesthesia management for patients who underwent appendectomy was carried out by anesthesia professionals using either GA or SA following a standardized protocol. Preoperative evaluation, documentation of preoperative vital signs, and premedication with intravenous dexamethasone were performed in the anesthesia waiting area. For patients receiving GA, the available analgesics (1.5 mg/kg diclofenac and 1-2 mg/kg pethidine) were administered. Patients were then preoxygenated for 3-5 minutes and induced under rapid sequence induction with Ketofol (ketamine-propofol in a 1:2 ratio) and suxamethonium at a dose of 2 mg/kg. Endotracheal intubation was then performed using a Macintosh-type laryngoscope. Following intubation, mechanical ventilation was initiated using a standard anesthesia machine, with a tidal volume of 7‐10 mL/kg and a respiratory rate of 10‐14 breaths per minute. Throughout the surgical procedure, anesthesia was maintained using halothane or isoflurane, intravenous normal saline was infused, and muscle relaxation was achieved using vecuronium. Intraoperative vital signs were continuously monitored and recorded at 5-minute intervals by the attending anesthetist until completion of the procedure. Upon completion of surgery, neuromuscular blockade was reversed with the administration of 0.05 mg/kg of neostigmine and atropine at a dosage of 0.2 mg/kg. Patients were then extubated in the operating theater before being transferred to the postanesthesia care unit (PACU).

For patients receiving SA, patients were positioned in a sitting position. Under complete aseptic technique, the skin was infiltrated with 3 mL of 2% lidocaine, and lumbar puncture was performed at the level of the L3-L4 lumbar interspace using a midline approach with a 24- or 25-gauge spinal needle. Once free flow of clear cerebrospinal fluid was restored, 3.5‐4.0 mL of hyperbaric bupivacaine was injected intrathecally, and the patient was then asked to lie in a supine position. The adequacy of anesthesia was assessed using the pinprick method for the sensory block level and the modified Bromage scale for the motor block level [27]. Surgery was started once the target sensory block level of the T6-T4 dermatomes was achieved. Throughout surgery, intravenous normal saline was infused, and intraoperative vital signs were continuously monitored and recorded at 5-minute intervals. Upon completion of the surgical procedure, patients were transferred to the PACU.

Postoperatively, data collectors documented the incidence of PONV in both the PACU and within the first 24 hours following PACU discharge. Pain intensity was assessed using an 11-point numeric rating scale (NRS; scores ranging from 0=“no pain” to 10=“worst imaginable pain”) at the first, second, sixth, 12th, and 24th postoperative hours. The cumulative consumption of diclofenac and tramadol, both in the PACU and within the first 24 hours postoperatively, was recorded. In addition, postoperative outcomes, including the occurrence of complications, time to first ambulation, time to first oral intake, and time to discharge home, were recorded.

Postoperative QoR was assessed 24 hours after surgery using the QoR-15 questionnaire. QoR-15 is a validated and reliable tool designed to assess patient-reported QoR in the immediate postoperative period. It assesses 5 dimensions of recovery: psychological support, physical comfort, emotional support, physical independence, and pain level. The tool comprises 15 items, each scored on an 11-point Likert scale (from 0=“strongly negative” to 10=“strongly positive”), resulting in a total score ranging from 0 to 150, with higher scores indicating better QoR [19,28].

### Data Quality Control

To assess the validity of the questionnaire, a pilot study was conducted on 20 participants at Debre Tabor Comprehensive Specialized Hospital, 1 month before the actual data collection period. Data collectors and supervisors were trained in the proper use of data collection tools and the overall data collection process. Throughout the data collection period, regular supervision and close follow-up were conducted to ensure data completeness, consistency, and accuracy.

### Statistical Analysis

Data were coded and entered using EpiData (version 4.6; EpiData Association) software and exported to and analyzed using Stata (version 17; StataCorp) software. The normality of continuous variables was assessed using the Shapiro-Wilk test. Categorical variables are presented as frequencies and percentages, while continuous variables are reported as either means with SDs or medians with IQRs, as appropriate. Intergroup comparisons between the SA and GA groups were performed using the chi-square test (or Fisher exact test, where appropriate) for categorical variables and the independent *t* test or Mann-Whitney *U* test for continuous variables, depending on the distribution of the data.

### Ethical Considerations

Ethics approval for the study was obtained from the research ethical review committee of the College of Health Sciences at Debre Tabor University (CHS 17/2017). The study was conducted in accordance with the Declaration of Helsinki, local legislation, and institutional requirements. Informed consent was obtained from each study participant, and confidentiality was ensured by avoiding questions that contained identifiers or sensitive content.

## Results

### Sociodemographic and Clinical Characteristics

A total of 74 participants, including 37 in the SA group and 37 in the GA group, were included in our study. Age, sex, BMI, and comorbid status were comparable between the 2 groups, with no statistically significant difference between them. Moreover, the mean duration of surgery, intraoperative blood loss, and amount of intraoperative fluid administered were comparable between the 2 groups (Table 1).

**Table 1.** Sociodemographic and clinical characteristics of study participants^a^

Variables		General anesthesia group (n=37)	Spinal anesthesia group (n=37)	*P* value
Age (years), mean (SD)^a^		27.6 (4.2)	29 (4.7)	.18
Sex, n (%)				.48
Female		17 (23)	14 (19)	
Male		20 (27)	23 (31)	
BMI (kg/m^2^), median (IQR)		20.1 (19.5-21.2)	20.5 (20.3-22.1)	.12
Comorbidities, n (%)				.76
No		31 (41)	30 (40)	
Yes		6 (8)	7 (9)	
American Society of Anesthesiologists status, n (%)				.76
I		31 (42)	30 (40)	
II		6 (8.1)	7 (9.5)	
Intraoperative blood loss (mL), median (IQR)		60 (50-80)	50 (50-80)	.08
Intraoperative fluid administered (mL), median (IQR)		700 (500-800)	600 (500-700)	.09
Duration of surgery (minutes), mean (SD)^a^		46.4 (8.7)	42.8 (8.9)	.09

a Values expressed as mean (SD) were analyzed using the independent *t* test, values expressed as frequencies (%) were analyzed using the chi-square test, and values expressed as median (IQR) were analyzed using the Mann-Whitney *U* test.

### Postoperative QoR

The total postoperative QoR-15 score at 24 hours was significantly higher in the SA group than in the GA group, with median scores of 128 (IQR 124-136) vs 100 (IQR 90-133), respectively (*P*<.001). A detailed analysis of individual QoR-15 items revealed that the SA group scored significantly higher on the majority of items than the GA group (Table 2).

**Table 2.** Comparison of general and spinal anesthesia on postoperative quality of recovery in patients after appendectomy.^a^

Variables	General anesthesia group	Spinal anesthesia group	*P* value^b^
Postoperative total Quality of Recovery-15 score, median (IQR)	100 (90-133)	128 (124-136)	<*.001*
Been able to enjoy food, mean (SD)	6.1 (1.9)	7.4 (1.4)	.*002*
Feeling rested, mean (SD)	7.7 (1.9)	8.4 (1.4)	.06
Able to look after personal toilet and hygiene unaided, mean (SD)	6.4 (1.1)	7.7 (1.5)	<*.001*
Getting support from hospital physicians and nurses, mean (SD)	9.5 (0.7)	9.3 (0.8)	.30
Able to return to work or usual home activities, mean (SD)	4.9 (1.6)	5.6 (1.2)	<*.001*
Moderate pain, mean (SD)	5.5 (2.0)	6.5 (1.4)	*.02*
Severe pain, mean (SD)	6.8 (1.8)	7.8 (1.4)	*.02*
Able to breathe easily, median (IQR)	8 (5-10)	10 (8-10)	*<.001*
Have had a good sleep, median (IQR)	6 (5-8)	9 (8-9)	*<.001*
Able to communicate with family or friends, median (IQR)	9 (9-10)	10 (10-10)	*.001*
Feeling comfortable and in control, median (IQR)	6 (6-9)	9 (8-10)	*<.001*
Having a feeling of general well-being, median (IQR)	8 (6-10)	9 (8-10)	*.004*
Nausea and vomiting, median (IQR)	10 (8-10)	10 (10-10)	*.003*
Feeling worried or anxious, median (IQR)	8 (7-10)	10 (9-10)	*<.001*
Feeling sad or depressed, median (IQR)	8 (6-10)	10 (8-10)	*.005*

a Values expressed as mean (SD) were analyzed using the independent *t* test, whereas values expressed as median (IQR) were analyzed using the Mann-Whitney *U* test.

b *P *values of <.05 are considered statistically significant and are italicized.

### Postoperative Complications and Outcomes

In terms of PONV, the incidence of nausea and vomiting in the PACU was significantly higher in the GA group than in the SA group (*P=.*02). However, the incidence of PONV within 24 hours after PACU discharge was comparable between the 2 groups (*P=.*67). In addition, postoperative complications, such as postoperative adhesions, surgical site infections, and relaparotomy, were not observed in either group. Pain scores on the NRS at the first, second, sixth, and 12th postoperative hours were significantly lower (*P<.*001) in the SA group than in the GA group, whereas pain scores at the 24th postoperative hour were comparable between the two groups (*P=.*12). Regarding the postoperative analgesic consumption, cumulative diclofenac and tramadol consumption in the PACU and within the first 24 postoperative hours were significantly higher in the GA group than in the SA group. Furthermore, the times to first ambulation, first oral intake, and discharge home were significantly lower in the SA group than in the GA group (*P<.*001, *P<.*001, and *P=.*005, respectively; Table 3).

**Table 3.** Comparison of secondary recovery outcomes of study participants between the general anesthesia (GA) and the spinal anesthesia (SA) groups.^a^

Variables		GA group (n=37)	SA group (n=37)	*P* value^b^
PONV^c^ in the PACU^d^, n (%)				*.02*
Yes		9 (12)	2 (3)	
No		28 (38)	35 (47)	
PONV within 24 postoperative hours, n (%)				.67
Yes		4 (5)	2 (3)	
No		33 (45)	35 (47)	
NRS^e^ score at first hour postoperatively, median (IQR)		3 (1-4)	0 (0-0)	*<.001*
NRS score at second hour postoperatively, median (IQR)		4 (3-6)	0 (0-0)	*<.001*
NRS score at sixth hour postoperatively, mean (SD)		4.2 (2.9)	2.1 (1.9)	*<.001*
NRS score at 12th hour postoperatively, mean (SD)		6 (1.4)	4.6 (0.9)	*<.001*
NRS score at 24th hour postoperatively, mean (SD)		5.5 (1.5)	4.9 (1.1)	.12
Total diclofenac consumption in PACU (mg), median (IQR)		75 (0-75)	0 (0-0)	*<.001*
Total diclofenac consumption within the first 24 hours (mg), median (IQR)		150 (75-150)	75 (75-150)	*<.001*
Total tramadol consumption in PACU (mg), median (IQR)		50 (0-50)	0 (0-0)	*<.001*
Total tramadol consumption within the first 24 hours (mg), mean (SD)		185.1 (10.4)	133.8 (13.1)	*.003*
Time to first ambulation (hours), median (IQR)		12 (10-13)	9 (8-10)	*<.001*
Time to first oral intake (hours), median (IQR)		13 (12-14)	9 (9-12)	*<.001*
Time to discharge (hours), mean (SD)		48.8 (6.2)	42.6 (10.9)	*.005*

a Values expressed as frequencies (%) were analyzed using the chi-square test (for PONV in the PACU) or the Fisher exact test (for PONV within 24 postoperative hours), values expressed as mean (SD) were analyzed using the independent *t* test, and values expressed as median (IQR) were analyzed using the Mann-Whitney *U* test.

b *P *values of <.05 are considered statistically significant and are italicized.

c PONV: postoperative nausea and vomiting.

d PACU: postanesthesia care unit.

e NRS: Numeric Rating Scale.

## Discussion

### Principal Findings

This prospective cohort study aimed to compare the postoperative recovery outcomes between patients who underwent appendectomy under SA and those who received GA. The findings revealed that patients in the SA group had significantly better early postoperative recovery profiles, as evidenced by higher QoR-15 scores, lower postoperative pain and analgesic consumption, reduced incidence of early PONV, and faster recovery outcome measures.

Our study revealed that patients who underwent appendectomy under SA experienced better recovery, with higher total postoperative QoR-15 scores and improved scores on the majority of individual QoR-15 items. These findings align with previous studies comparing neuraxial anesthesia with GA, which also showed enhanced recovery profiles in patients receiving neuraxial techniques [18,26,29]. One possible explanation for this finding is that patients receiving GA are exposed to multiple systemic medications, including volatile agents and opioids, which may have residual postoperative effects and impair early postoperative recovery [30]. In contrast, neuraxial techniques such as SA provide more effective pain control, reduce the need for systemic medications, and minimize opioid-related side effects, thereby contributing to enhancing overall well-being and recovery [29].

Supporting the QoR findings, postoperative pain management outcomes were significantly improved in the SA group during the early postoperative hours. Pain scores were significantly lower at the first, second, sixth, and 12th postoperative hours. This can be attributed to SA’s ability to block the afferent nociceptive pathway, thereby providing long-lasting postoperative pain relief despite recovery of motor function [30]. This superior quality of postoperative analgesia was directly related to a significant reduction in analgesic requirements, which may have contributed to the improved QoR-15 scores and faster mobilization [31]. At the 24th postoperative hour, pain scores were comparable between the 2 groups. This may be due to the wearing-off effects of SA and the initiation of standard oral analgesia protocols. In addition, both cumulative diclofenac and tramadol consumption in both the PACU and during the first 24 postoperative hours was significantly lower in the SA group. The reduction in postoperative opioid requirement is particularly important because it mitigates opioid-related side effects such as nausea, vomiting, sedation, and ileus [32,33].

In terms of clinical significance, between-group differences in pain scores were most pronounced during the first 6 hours, with differences of approximately 3 points at 1 hour, 4 points at 2 hours, and 2.1 points at 6 hours, which are generally considered clinically meaningful. In contrast, the difference observed at 12 hours (1.4 points) was smaller and may be of borderline clinical relevance, whereas no clinically significant difference was detected at 24 hours [34]. When analgesic consumption was expressed in intravenous morphine equivalents, the reduction in 24-hour opioid use corresponds to approximately 5 mg of intravenous morphine, which falls below commonly accepted thresholds for a clinically important effect. This finding suggests that, despite statistical significance, the overall reduction in opioid requirements over 24 hours was modest, and the clinical importance of this difference should be interpreted with caution [35].

In this study, the incidence of PONV in the PACU was significantly higher in the GA group. This may be due to the residual effects of inhaled anesthetic agents and increased opioid use for postoperative analgesia, which are known risk factors for PONV [6,36,37]. The incidence of PONV after discharge from the PACU was similar in both groups, which may reflect the resolution of early anesthetic effects and the standardization of postoperative care.

In addition, functional recovery outcome measures, such as time to first ambulation, time of first oral intake, and time of discharge to home, were significantly better in the SA group. Better pain control, reduced sedative drug requirements, and fewer side effects in the SA group may contribute to a quicker return to baseline function, reduced hospital stay, and lower health care costs [17,38]. Conversely, the use of inhaled anesthetics, sedatives, and opioids in the GA group may contribute to postoperative complications, such as cardiorespiratory depression, delayed bowel activity, and paralytic ileus, which can delay oral intake and ambulation and prolong hospitalization [6].

This study has some limitations. First, the study was conducted at a single center, and the generalizability of the findings to a wider population might be limited. In addition, although the sample size was determined using appropriate statistical methods, the final number of participants included in each group was relatively small. This may have reduced the statistical power of the study and may also restrict the external validity of the findings. Second, the study participants were selected in a nonrandomized manner, and allocation of anesthesia types may have introduced selection bias. Finally, this study focused exclusively on immediate QoR and did not include intermediate- and long-term follow-up, which limits conclusions regarding the intermediate- and long-term recovery outcomes.

### Conclusions and Recommendations

The findings of our study demonstrated that SA provides better early postoperative recovery and postoperative analgesia than GA in patients undergoing appendectomy. In addition, patients undergoing appendectomy under SA had reduced early postoperative analgesia consumption, a reduced incidence of early PONV, and faster in-hospital recovery trajectories than those undergoing appendectomy under GA. Therefore, in the absence of specific contraindications, SA seems to be a preferred anesthesia technique for patients undergoing appendectomy for acute appendicitis. We recommend that future research explore intermediate- and long-term recovery outcomes in patients who undergo appendectomy under SA vs GA.

## Abbreviations

GA: general anesthesia
PACU: postanesthesia care unit
PONV: postoperative nausea and vomiting
QoR: quality of recovery
QoR-15: Quality of Recovery-15
SA: spinal anesthesia
